# Purkinje cell stripes and long-term depression at the parallel fiber-Purkinje cell synapse

**DOI:** 10.3389/fnsys.2014.00041

**Published:** 2014-03-28

**Authors:** Richard Hawkes

**Affiliations:** ^1^Department of Cell Biology and Anatomy, University of CalgaryCalgary, AB, Canada; ^2^Hotchkiss Brain Institute, University of CalgaryCalgary, AB, Canada; ^3^Genes and Development Research Group, Faculty of Medicine, University of CalgaryCalgary, AB, Canada

**Keywords:** zebrin II, phospholipase Cβ4, Purkinje cell, stripes, long-term depression

## Abstract

The cerebellar cortex comprises a stereotyped array of transverse zones and parasagittal stripes, built around multiple Purkinje cell subtypes, which is highly conserved across birds and mammals. This architecture is revealed in the restricted expression patterns of numerous molecules, in the terminal fields of the afferent projections, in the distribution of interneurons, and in the functional organization. This review provides an overview of cerebellar architecture with an emphasis on attempts to relate molecular architecture to the expression of long-term depression (LTD) at the parallel fiber-Purkinje cell (pf-PC) synapse.

The general hypothesis explored in this review is that the elaborate molecular architecture of the cerebellar cortex has its counterpart in the compartmentation of function. In particular, many forms of synaptic plasticity have been identified in the cerebellar cortex (e.g., Hansel et al., 2001)—a network property that De Zeeuw et al. have called “distributed synergistic plasticity” (Gao et al., 2012). Both long-term depression (LTD) and long-term potentiation (LTP) have been identified, and these are expressed at multiple synapses—parallel-fiber to Purkinje cell, mossy fiber to granule cell, inhibitory interneuron to Purkinje cell (“rebound potentiation”: e.g., Tanaka et al., 2013) etc.

By way of example, the review focuses on LTD at the parallel fiber-Purkinje cell (pf-PC) synapse. A brief consideration of its opposite –LTP—is also included. Other forms of Purkinje synaptic plasticity in the cerebellum are not included since so little is known of their relationship to the stripe architecture. Therefore, to set the stage the review begins with a brief overview of the patterning of the main players—Purkinje cells, climbing and mossy fiber afferents, and granule cells.

## Overview of zone and stripe architecture

### Purkinje cells

**Figure 1:**
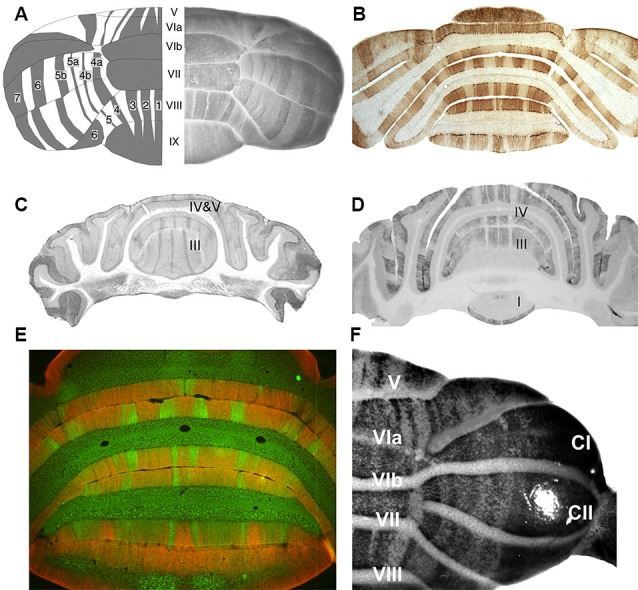
**Stripes in the adult mouse cerebellar cortex as revealed by various Purkinje cell subset markers**. **(A)** On the right is a whole mount dorsal view of a hemicerebellum immunoperoxidase stained for zebrin II/aldolase C. On the left is a cartoon view: lobules are numbered with Roman numerals (V–IX); zebrin II+ stripes as 1–7 (Adapted from Furutama et al., 2010). **(B)** A transverse section through the posterior lobe immunoperoxidase stained by using anti-zebrin II (Adapted from Marzban et al., 2004). **(C)** A transverse section through the anterior lobe immunoperoxidase stained for phospholipase Cβ3 (PLCβ3) (Adapted from Sarna et al., 2006). **(D)** A transverse section taken close to that in panel C, immunoperoxidase stained for PLCβ4 (Adapted from Sarna et al., 2006). **(E)** Transverse section through the posterior lobe double immunofluorescence labeled for GABA type B receptors 2 (GABA_B_R2) (red) and PLCβ4 (green) (Adapted from Chung et al., 2008). **(F)** A whole mount dorsal view of a hemicerebellum from an IP_3_R1nls-lacZ transgenic mouse X-gal stained for transgene expression (Adapted from Furutama et al., 2010).

Several recent reviews have described the architecture of the adult cerebellar cortex (e.g., Apps and Garwicz, 2005; Apps and Hawkes, 2009; Ruigrok, 2011). In brief, a range of expression markers expressed in subsets of Purkinje cells have revealed an orthogonal matrix of transverse zones and parasagittal stripes (Figure 1). First, the cerebellar cortex is divided by transverse boundaries into transverse zones. These are most easily recognized in the vermis but appear to have their counterparts in the hemispheres as well. Each transverse zone is further subdivided into long narrow stripes that run parasagittally from rostral to caudal. The most-studied example is the expression pattern of zebrin II/aldolase C, which identifies a stereotyped array of zebrin II+ and zebrin II- stripes (e.g., Brochu et al., 1990; Hawkes and Gravel, 1991; Ahn et al., 1994; Hawkes and Herrup, 1995; Figures 1A, B). The combination of multiple such patterns adds up to a cerebellar cortex with several hundred distinct topographical units (e.g., Hawkes, 1997; Hawkes et al., 1997, 1999; Armstrong et al., 2000).

The Purkinje cell expression domains are reproducible between individuals to a remarkable level—individual stripes comprised of no more than 100 or so Purkinje cells are faithfully reproduced (e.g., the P4b+/P5a+ stripes in the hemispheres: Hawkes and Leclerc, 1987; Figures 1A, B). Indeed, although the size of a particular zone or stripe may be modified to suit the animal’s mode of life a common ground plan is conserved across all mammals studied to date (~30 species—Sillitoe et al., 2005; Marzban and Hawkes, 2011) and is also found in birds (Pakan et al., 2007; Iwanuik et al., 2009; Marzban et al., 2010).

### Afferent projections

Stripes of Purkinje cells are targets of specific afferent subsets during development and restrict their terminal fields in the adult, with the result that specific afferent subsets terminate in stripes. Studies over the past 25 years or so have shown that afferent terminal fields are precisely aligned with Purkinje cell stripes. These studies have combined immunocytochemistry for stripe antigens with anterograde tracing to generate detailed topographical maps, in particular relating afferent terminal fields to zebrin II+/− stripes (e.g., climbing fibers—Gravel et al., 1987; Voogd et al., 2003; Sugihara and Shinoda, 2004; Voogd and Ruigrok, 2004; Sugihara and Quy, 2007 etc.; mossy fibers—Gravel and Hawkes, 1990; Akintunde and Eisenman, 1994; Ji and Hawkes, 1994; Armstrong et al., 2009; etc.). In some cases, molecular differences have also been demonstrated between afferent subsets. For example, mossy fibers that express somatostatin terminate on Purkinje cell stripes that express the small heat shock protein (HSP25; Armstrong et al., 2009), and climbing fibers immunoreactive for corticotropin-releasing factor (CRF) terminate selectively on zebrin II+ Purkinje cells (Sawada et al., 2008; see Section Corticotropin-releasing Factor).

Although striped patterns of Purkinje cell gene expression are aligned with stripes of afferent innervation, the formation and maintenance of stripes is not contingent upon afferent input: chemical or surgical afferent lesions do not alter the pattern (zebrin—Leclerc et al., 1988; Zagrebelsky et al., 1996, 1997; sphingosine kinase 1a—Terada et al., 2004; HSP25—Armstrong et al., 2001; L7/pcp2—Oberdick et al., 1993; etc.), and subtype phenotypes are expressed in slice and dissociated cerebellar cultures and after grafting the cerebellar anlage to an ectopic location (e.g., Wassef et al., 1990; Seil et al., 1995).

### Granule cells

Purkinje cell stripe boundaries are also restriction boundaries for interneurons. Most prominent among these are the granule cells. First, the analysis of murine chimeras has identified a reproducible set of lineage boundaries within the granular layer that align with the transverse boundaries seen in the Purkinje cells (Hawkes et al., 1999). Multiple expression boundaries are also found at these locations in the adult and in the external granular layer during development (reviewed in Armstrong and Hawkes, 2000; Consalez and Hawkes, 2013). This strongly suggests that different granule cell lineages exploit the underlying Purkinje cell zonal architecture as the external granular layer spreads to cover the embryonic cerebellar anlage. Secondly, in the adult granular layer a complex array of patches and stripes can be revealed (e.g., nitric oxide (NO) synthase or its surrogate, reduced nicotinamide adenine dinucleotide phosphate (NADPH) diaphorase: Hawkes and Turner, 1994; Schilling et al., 1994; Ozol and Hawkes, 1997; Hawkes et al., 1998). These also align with the Purkinje cell architecture. It is difficult to credit that these represent cell autonomous properties of the granule cells, given the challenges such a model would present for the targeting of granule cell migration and settling, so it is more likely that the expression patterns are secondary to the local environment (e.g., Purkinje cells or mossy fibers).

## Functional correlates of stripes

Given that pretty much everything in the anatomy of the cerebellar cortex is stripy, it should be unsurprising that similar compartmentation is seen by using functional mapping. First, parasagittal stripes are seen in electrophysiological recordings from the cerebellar cortex—the 12 A-D2 longitudinal zones and microzones (e.g., Oscarsson, 1979; for an account of the baroque terminology of cerebellar architecture, see Apps and Hawkes, 2009)—and these align with, and are likely the same thing as, the striped domains of differential gene expression. Similarly, optical imaging of the cerebellar cortex also reveals a parasagittally striped functional organization (e.g., Chen et al., 1996; Ebner et al., 2005, 2012; Gao et al., 2006). In contrast, recordings of tactile receptive fields in the hemispheres apparently reveal a somewhat different organization—a complex but reproducible array of functional patches responsive to different stimulus sites—vibrissae, lips, teeth etc., (“fractured somatotopy”: reviewed in Welker, 1987). However, when the tactile receptive field boundaries and antigenic boundaries are compared, a reproducible alignment is found (e.g., Chockkan and Hawkes, 1994; Hallem et al., 1999) that is consistent with the evidence cited above that mossy fiber afferent terminal fields show stripe restriction.

Different Purkinje cell stripes receive climbing fibers from different sources. Consistent with this topography, Paukert et al. (2010) recently showed that climbing fibers that terminate on zebrin II+ Purkinje cells release more glutamate per action potential than do those terminating in zebrin II− stripes. As a result, climbing fiber-mediated excitatory postsynaptic potentials in Purkinje cells decay more slowly in the zebrin II+ stripes, and thus longer-duration complex spikes are triggered. The implication is that prolonged climbing fiber-induced depolarization of Purkinje neurons in zebrin II+ stripes should preferentially enhance Ca^2+^ influx and thereby facilitate activity-dependent changes in the strength of both climbing and parallel fiber synapses (Hansel et al., 2001; Safo et al., 2006; Carey and Regehr, 2009; Mathy et al., 2009).

Finally, Wadiche and Jahr (2001, 2005) have shown that Purkinje cells in zebrin II+/− stripes express different complements of excitatory amino acid transporters (EAATs), some of which are more effective than others. As a result, regional differences in glutamate transporter expression affect the degree of metabotropic glutamate receptor (mGluR1) stimulation (see Section Glutamate Re-uptake).

## Molecular corelates of long-term depression at the parallel fiber-purkinje cell synapse

The functional differences between stripes derive in two ways. On the one hand they reflect differences in connectivity (i.e., the striped organization of the olivocerebellar and mossy fiber projections). On the other hand—and central to what follows—different stripes display distinctly different intrinsic properties, notably a variety of different forms of synaptic plasticity (e.g., reviewed in Hansel et al., 2001). The hypothesis explored in this review is that the specificity of the afferent topography together with the molecular heterogeneity of the granule cells and Purkinje cells constitutes a substrate for multiple plastic adaptations of the pf-PC synapse. What follows focuses on LTD at the pf-PC synapse as an exemplar.

Purkinje cells receive 2 glutamatergic excitatory inputs, one from mossy fibers via pf-PC synapses on dendritic spines and another from climbing fibers onto the dendritic shafts. Conjunctive stimulation of the parallel fiber and climbing fiber pathways (1–4 Hz for 1–10 min) results in a long-lasting depression of transmission at the pf-PC synapse (e.g., recently reviewed in Vogt and Canepari, 2010; Finch et al., 2012; an excellent history is provided in Kano et al., 2008). LTD has often been evoked as a model of cerebellar motor learning, but recent studies cast doubt on this (Schonewille et al., 2011; Gao et al., 2012).

LTD at the pf-PC synapse is quantitatively different between stripes: it is easier to induce pf-PC synapse LTD in zebrin II- than in zebrin II+ Purkinje cells (Wadiche and Jahr, 2001). Little is known of the molecular basis for differences in LTD across stripes but it is striking that many molecules whose expression is in stripes are associated with the putative pathways leading to LTD (Table 1).

**Table 1:** **A list of the synaptic molecules with striped expression patterns referred to in the text; whether they are preferentially expressed in zebrin II+ (zII+) or zebrin II− (zII−) stripes (or a mixture of both); and pertinent citations**.

**Molecule**	**Stripe Preference**	**Citations**
Synaptophysin	zII+/zII−	Hawkes et al., 1985; Leclerc et al., 1989
Dysbindin	zII+/zII−	Sillitoe et al., 2003
nNOS/NADPHd	zII+/zII−	Yan et al., 1993; Hawkes and Turner, 1994; Schilling et al., 1994; Baader and Schilling, 1996
Neuroplastin	zII−	Marzban et al., 2003
mGluR1b	zII−	Mateos et al., 2001
EAAT4	zII+	Dehnes et al., 1998
NMDA receptor (NR2C^nlacZ^)	zII−	Karavanova et al., 2007
CRF	zII+	Sawada et al., 2008
PLCβ3	zII+	Sarna et al., 2006
PLCβ4	zII−	Sarna et al., 2006
IP_3_R-nls-LacZ	zII+	Furutama et al., 2010
PKCδ	zII+	Barmack et al., 2000
GABABR2	zII+	Albin and Gilman, 1989; Luján and Shigemoto, 2006; Chung et al., 2008
Neurogranin	zII−	Larouche et al., 2006
PEP-19	?	Wassef et al., 1992

**Figure 2:**
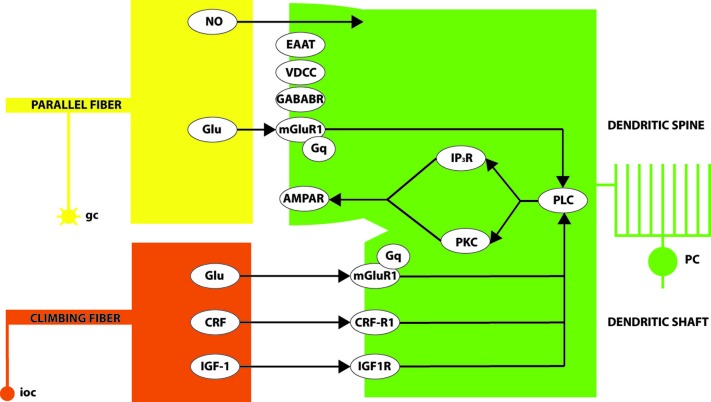
**A simplified model of some of the signaling pathways leading to LTD of the pf-PC synapse**. Conjunctive glutamate (Glu) release at the granule cell (gc)/parallel fiber synapse on the Purkinje cell (PC) dendritic spine and the climbing fiber (from inferior olivary cells: ioc) synapses on the dendritic shaft activates the metabotropic glutamate receptor (mGluR1). Glutamate signaling across the synaptic cleft is modulated by excitatory amino acid transporters (EAAT). A signaling pathway via Gq proteins activates PLC. In turn, PLC signals via both the inositol triphosphate receptor (IP_3_R) and protein kinase C (PKC). The upshot is the internalization of synaptic AMPA receptors and consequent LTD. The overall process is also modulated by various other signals including: presynaptic nitric oxide release (NO); the binding of corticotropin-releasing factor (CRF) to its receptor (CRF-R1) and insulin-like growth factor (IGF)-1 binding to its receptor (IGF-1R), both of which signal via PLC; signaling via postsynaptic GABA_B_R; and calcium influx through voltage-dependent calcium channels (VDCCs).

An influential model of the molecular basis of LTD at the pf-PC synapse, due to Ito (e.g., reviewed in Ito, 1984, 1989, 2002), is summarized in a simplified fashion in Figure 2. In brief, conjunctive glutamate release from parallel fiber and climbing fiber terminals acts through mGluR1 to activate several parallel intracellular signaling pathways—in particular, one via phospholipase C (PLC) and diacylglycerol (DAG) to protein kinase C (PKC), and another via inositol triphosphate (IP_3_). The downstream consequence is the internalization of AMPA (α-amino-3-hydroxy-5-methyl-4-isoxazolepropionic acid)-sensitive glutamate receptors (AMPAR), and consequent synaptic desensitization. NO and CRF play supporting roles. It is instructive to examine the expression patterns of the different players in this pathway.

### Synaptic markers

First of all a reproducible pattern of stripes is revealed in the molecular layer of the cerebellar cortex by using immunocytochemistry for the synaptic vesicle protein synaptophysin (Hawkes et al., 1985), with stripes of higher expression alternating with those of lower expression, both in the granular layer associated with mossy fiber synaptic glomeruli and in the molecular layer, associated primarily with pf-PC synapses (Hawkes et al., 1985; Leclerc et al., 1989). The interpretation of the observation is less obvious—does stronger staining reflect more antigen per vesicle, antibody access, more vesicles per synapse, a higher Purkinje cell spine density …? Appropriate comparisons with the expression patterns of other synaptic markers that might resolve this question have not been reported. A patchy/striped arrangement of mossy fiber terminals in the granular layer is reported with other presynaptic markers, but in these cases the expression in the molecular layer appears to be uniform (e.g., dysbindin—Sillitoe et al., 2003; neuronal nitric oxide synthase (nNOS); see Section Nitric Oxide). A uniform distribution in the molecular layer may be misleading in that differential expression of granule cell markers is easier to discern in the granular layer, where the somata are segregated into stripes and clusters, than in the molecular layer where the long parallel fiber trajectories extensively overlap and smooth out different expression levels from different granule cell populations.

Structural synaptic proteins are also differentially expressed. For example, a prominent striped expression pattern is revealed by immunocytochemical staining for the postsynaptic membrane glycoprotein neuroplastin (Marzban et al., 2003; note—in the hippocampus, neuroplastin has been linked to the inhibition of LTP; Empson et al., 2006). High levels of neuroplastin expression are preferentially associated with the zebrin II- Purkinje cell stripes.

### Glutamate receptors

Glutamate released into the synaptic cleft at both the climbing fiber and the pf-PC synapse binds to three types of glutamate receptor in the postsynaptic membrane—metabotropic (mGluR), GluRδ2 and AMPAR. mGluRs are G protein-coupled receptors with 7 transmembrane segments that do not form ion channels but rather signal via intracellular chemical messenger systems. Eight genes coding for different subtypes of mGluRs have been identified, seven of which are expressed in the cerebellum. In particular, Purkinje cell mGluR1 is localized in the peri- and extra-synaptic membranes. It is functionally coupled to PLC through which it modulates the IP_3_ (1,4,5)/Ca2+ signaling pathway and plays a key role in the induction of pf-PC LTD (reviewed in Knöpfel and Grandes, 2002).

There are at least 4 mGluR1 splice variants with differing subcellular and cellular distributions (mGluR1a-d: e.g., Conn and Pin, 1997). In the case of mGluR1b in the cerebellum, expression is striped in the molecular layer and co-located with zebrin II- stripe markers (Mateos et al., 2001). mGluR1a is also located in the Purkinje cell dendritic spine (e.g., Mateos et al., 2000) but whether or not there is an mGluR subtype restricted to the zebrin II+ Purkinje cell dendritic spines is not known.

GluRδ2 is also highly expressed in cerebellar Purkinje cells and is localized specifically to pf-PC synapses (Araki et al., 1993; reviewed in Hirano, 2006). GluRδ2 neither binds glutamate nor conducts current but rather regulates mGluR1-mediated synaptic transmission via PKCγ (e.g., Kato et al., 2012). Loss-of-function mutations in GluRδ2 result in multiple defects including impairment of LTD (Kashiwabuchi et al., 1995). There is no evidence that its expression is stripe-restricted (gain-of-function mutation of the GluRδ2 gene in the *lurcher* mouse (*Grid^Lc^/+*) results in striped Purkinje cell degeneration (Zuo et al., 1997; reviewed in Armstrong et al., 2011) but this likely reflects differential sensitivity to the insult rather than selective GluRδ2 expression).

### Glutamate re-uptake

The time that glutamate resides in the synaptic cleft, and hence is available for receptor binding, is governed by EAAT. In particular, EAAT4 has been implicated at the pf-PC synapse. It is therefore striking that the expression of EAAT4 is different from stripe to stripe with high levels associated with the zebrin II+ stripes (Dehnes et al., 1998). As a result, regional differences in glutamate transporter expression affect the degree of mGluR1 receptor stimulation, with the result that pf-PC LTD is dampened in Purkinje cells expressing high levels of EAAT4 (= zebrin II+; Wadiche and Jahr, 2005).

### Calcium influx

One consequence of mGluR1 activation is Ca^2+^ influx via VDCC in the postsynaptic dendritic membrane. There is no evidence that VDCCs are expressed differentially by Purkinje cell subsets (As for the *lurcher* mouse, it has been shown that a mutation of the VDCCα1a channel in the *tottering* mouse (*Cacna1a^tg^*) results in the selective Purkinje cell death of the zebrin II- Purkinje cell subset, but again the evidence suggests that this is due to differential sensitivity to an abnormal Ca^2+^ influx rather than restricted expression of the α1a channel; Fletcher et al., 1996).

The downstream response to dendritic Ca^2+^ influx is modulated by calpacitins, notably the 2 Purkinje cell proteins PEP-19 and neurogranin/RC3. Sutcliffe and colleague have proposed that calpacitins regulate calmodulin availability in dendritic spines and thus regulates their ability to amplify the mobilization of Ca^2+^ in response to metabotropic glutamate receptor stimulation, releasing calmodulin rapidly in response to large influxes of Ca^2+^ and slowly in response to small increases. This action is inhibited by PKC-mediated phosphorylation (reviewed in Gerendasy and Sutcliffe, 1997; Díez-Guerra, 2010). α-Calmodulin kinase KII is also shown to be required for LTD at the pf-PC synapse (Hansel et al., 2006). Neurogranin knockout mice show deficits in the induction of hippocampal LTP (e.g., Pak et al., 2000) but no cerebellar phenotype is reported. On the other hand, the much more abundant PEP19 is directly implicated: in the PEP19 null mouse both motor learning and pf-PC LTD are impaired (Wei et al., 2011). During cerebellar development, both PEP19 (Wassef et al., 1992) and neurogranin (Larouche et al., 2006) expression is restricted to Purkinje cell subsets. However, in the adult PEP19 expression is uniformly expressed by all Purkinje cells (Mugnaini et al., 1987) whereas neurogranin has disappeared (Larouche et al., 2006), so any significance for patterned LTD at the adult pf-PC synapse is doubtful.

### Nitric oxide

In addition to releasing glutamate, parallel fibers also release NO. NO acts through inhibition of protein phosphatases in the Purkinje cell dendritic spine and thus enhance AMPAR phosphorylation. LTD is abolished in transgenic mice lacking nNOS (Lev-Ram et al., 1997). There is clear evidence of different stripes of nNOS in the granular layer of the cerebellar cortex (e.g., Yan et al., 1993; Hawkes and Turner, 1994; Schilling et al., 1994; Baader and Schilling, 1996). Similar striping is harder to discern in the molecular layer, perhaps obscured by the overlapping parallel fiber populations. The nNOS pathway is activated via N-methyl-D-aspartate (NMDA)-type glutamate receptors located at the pf-PC synapse (and/or located in the presynaptic terminals of inhibitory interneurons; Shin and Linden, 2005). Functional NMDA receptors are also expressed at climbing fiber-PC synapses, and channel blocking inhibits LTD (Piochon et al., 2010).

It is noteworthy that NMDA receptor expression, as revealed by an NRC2 subunit knock-in mouse (NR2C^nlacZ^), reveals stripes of granule cells similar to those revealed by PLCβ4 expression (= zebrin II-; Karavanova et al., 2007). There is no evidence that Purkinje cell NMDA receptors are expressed in stripes.

### Corticotropin-releasing factor

Glutamate release and binding to mGluR1 is also the first step in signaling via the climbing fiber pathway. In parallel to glutamate release, climbing fibers also secrete CRF (Barmack and Young, 1990), which plays a permissive role in LTD that is probably mediated through PKC (Miyata et al., 1999). However, not all climbing fibers express CRF. Whole mount immunocytochemistry shows that CRF is restricted to (or is expressed at higher levels in) a striped subset of climbing fiber terminals that terminate in zebrin II+ Purkinje cell stripes (mouse—Sawada et al., 2008). However, the significance of this may not be straightforward as previous studies reported uniform CRF expression (e.g., cat—Cummings, 1989) or expression differences between lobules but not in the form of stripes (e.g., developing mouse—Overbeck and King, 1999). There is no evidence that G-protein coupled CRF receptor (CRFR1) expression is similarly striped (e.g., Allen Brain Atlas).

### Insulin-like growth factor 1

As well as releasing CRF, climbing fiber synapses also store and release IGF-1 (Torres-Aleman et al., 1994). It is not known if IGF-1 or its receptor tyrosine kinase (IGF1R) is expressed in stripes in the adult cerebellum (in general in the brain IGF1R is broadly expressed—it is the ligands that show regional restriction: e.g., reviewed in D’Ercole et al., 1996). During early postnatal development, IGF-1 is also expressed in a zebrin II- Purkinje cell subset, where it acts to block apoptosis (Croci et al., 2011), but it is unclear whether selective expression is retained in the adult.

### Phospholipase Cβ

mGluR1 signals via the Gq subclass of G-proteins to PLCβ (reviewed in Knöpfel and Grandes, 2002). There are four PLCβ isoforms, encoded by distinct genes (PLCβ1-4; Bahk et al., 1994). Strikingly, PLCβ3 and PLCβ4 are expressed by distinct, non-overlapping subsets of Purkinje cells. PLCβ3 is confined to the zebrin II+ Purkinje cell subset (Figure 1C) and PLCβ4 expression is coextensive with the zebrin II- Purkinje cell subset (Figures 1D, E; Sarna et al., 2006; Marzban et al., 2007) (Unexpectedly, a small subset of zebrin II+ Purkinje cell stripes in the nodular zone of the mouse cerebellum (~ lobules IX and X)—those that express HSP25—is reproducibly immunonegative for both PLCβ3 and PLCβ4 (Sarna et al., 2006)—the implication of this is unclear).

### Phospholipase A

Parallel to the PLCβ pathway, there is also a signaling pathway via phospholipase A (PLA)—in particular, the PLA2 isoform: e.g., Linden, 1995; Le et al., 2010), which acts to break down phospholipids into arachidonic acid, a potent activator of PK Cγ (e.g., Shearman et al., 1989). At least 20 PLA2 isoforms have been identified, three of which have been reported in Purkinje cells (cPLA2α, sPLA2IIA, and iPLA2; Shirai and Ito, 2004). There is no evidence that any of these is restricted to a particular Purkinje cell subset.

### Inositol (1,4,5) triphosphate release

Two signaling pathways leading to LTD lie downstream of PLC. The first involves IP_3_ release from intracellular stores to bind to its receptor on the endoplasmic reticulum (IP_3_R; e.g., Furuichi et al., 1989; Maeda et al., 1989). The cerebellar distribution of IP_3_R has recently been reported in a transgenic mouse in which the IP_3_R promoter was fused to a β-galactosidase reporter and a nuclear localization signal (IP_3_R1nls-*lacZ*; Furutama et al., 1996, 2010; Figure 1F). Transgene expression in the heterozygote reveals a striking array of Purkinje cell stripes that can be traced continuously through embryogenesis through to adulthood. In general, IP_3_R1nls-l*acZ* transgene expression is restricted to the zebrin II+ Purkinje cell subset. The extent to which this distribution reflects any feature of the true restriction of the receptor—perhaps developmentally—or is a transgene artifact (due to the transgene insertion, promoter truncation, enhancer trapping, etc.), is questionable. Immunocytochemistry with antibodies against IP_3_R do not show Purkinje cell stripes (e.g., Mignery et al., 1989).

### Protein kinase c (PKC)

An additional second-messenger signaling pathway between glutamate release and the induction of LTD goes via the generation of DAG by PLC, which in turn activates PKC (Crépel and Krupa, 1988; for a general review of PKC, see Newton, 1995). There are seven PKC subtypes—three (α, β and γ) activated in a Ca^2+^/DAG-dependent manner and 4 (δ, Є, η and θ) Ca^2+^-independent (reviewed in Tanaka and Nishizuka, 1994). Activation of Ca^2+^-dependent PKC is necessary for induction of LTD at the pf-PC synapse (e.g., Ito, 1989, 2002; Daniel et al., 1998). LTD induction at the pf-PC synapse is blocked by the intracellular application of PKC inhibitors in Purkinje cells (Linden and Connor, 1991). De Zeeuw et al. (1998) constructed a transgenic mouse in which a Purkinje cell-specific promoter (pcp2-L7) was used to target the expression of a broad-spectrum PKC inhibitor (the pseudosubstrate PKC[19–31]) and thereby showed that PKC activation in the Purkinje cell is a prerequisite for the induction of LTD. None of the Ca^2+^/DAG-dependent PKC isoforms is expressed selectively by a Purkinje cell subset (e.g., Barmack et al., 2000).

The role(s), if any, of the four Ca^2+^-independent PKCs in LTD induction is unclear. However, they deserve attention here because while most PKC isoform distributions are uniform across the molecular layer the one exception is PKCδ, whose expression in the nodular zone of the rat reveals a reproducible striped expression pattern with higher levels in the zebrin II+ stripes (Barmack et al., 2000). Furthermore, experimental manipulation of the cerebellar afferent inputs by labyrinthectomy demonstrated an activity-dependent targeting of the PKCδ isoform to the pf-PC synapse (Barmack et al., 2001). PKCδ has been implicated in hippocampal LTP (e.g., Kim et al., 2013) but no specific role in cerebellar LTD is known.

### GABA_B_ receptors

LTD at the pf-PC synapse is also modulated by an unusual form of γ-aminobutyric acid (GABA) receptor signaling. In the adult cerebellum GABA_B_Rs are predominantly located perisynaptically at the dendritic spines of Purkinje cells (e.g., Turgeon and Albin, 1993; Kaupmann et al., 1997; Bischoff et al., 1999; Kulik et al., 2002; Fritschy et al., 2004; Luján and Shigemoto, 2006). Both GABA_A_ (reviewed in Fritschy and Panzanelli, 2006) and GABA_B_ receptor classes are expressed in the cerebellum but only GABA_B_Rs have been implicated in pf-PC LTD. GABA_B_Rs are G-protein-coupled receptors formed as heteromers of 2 subunits (GABA_B_R1/2; Marshall et al., 1999; Möhler and Fritschy, 1999). Postsynaptic GABA_B_R signaling enhances LTD (Tabata et al., 2004; Kamikubo et al., 2007), possibly as a Ca^2+^-dependent cofactor of mGluR1 signaling. The role of GABA_B_Rs in the modulation of LTD is unconventional in that it does not require GABA. Rather, extracellular Ca^2+^ binds to the GABA_B_R and constitutively increases the glutamate sensitivity of mGluR1 (Tabata et al., 2004). The role of GABA_B_R in LTD is relevant for the present discussion because immunocytochemistry for GABA_B_R2 shows a strong restriction of receptor immunoreactivity to the zebrin II+ stripes (Albin and Gilman, 1989; Luján and Shigemoto, 2006; Chung et al., 2008; Figure 1E).

### AMPA receptors

The ultimate downstream target of PLC signaling, via both PKC and IP_3_R, is the phosphorylation (via both PKC and Src-family protein tyrosine kinases—e.g., Tsuruno et al., 2008) of postsynaptic AMPA receptors (AMPAR; Ito, 1984; Crépel and Krupa, 1988; Hirano, 1991; Linden et al., 1991; Matsuda et al., 2000; Tsuruno et al., 2008; etc.). AMPAR kinetics, agonist affinity and unitary conductances are unchanged by phosphorylation (Linden, 2001) but rather there results a reduction in AMPAR number due to enhanced endocytosis (Matsuda et al., 2000), which is dependent on phosphorylation at ser-880 in the AMPAR GluR2 subunit (Chung et al., 2003; reviewed in Shin and Linden, 2005). There is no evidence of selective expression of either Src kinases or AMPAR by Purkinje cell subsets.

## Molecular corelates of long-term potentiation at the parallel fiber-purkinje cell synapse

The opposite process—LTP—countermands LTD at the pf-PC synapse. This endows the synapse with bidirectional plasticity (Lev-Ram et al., 2002; Coesmans et al., 2004). Postsynaptic LTP is induced by parallel fiber stimulation (1 Hz for 5 min: Lev-Ram et al., 2002, 2003). The signaling pathways implicated resemble those previously identified for hippocampal LTP (e.g., Jörntell and Hansel, 2006). Stimulation causes Ca^2+^ influx via voltage-sensitive channels, which activates several calmodulin-activated protein phosphatases (PP1, PP2A and PP2B; Lev-Ram et al., 2003; Coesmans et al., 2004; Belmeguenai and Hansel, 2005; Schonewille et al., 2010). In turn, this results in enhanced AMPA receptor insertion into the postsynaptic membrane (a process dependent upon NO—Huang et al., 2005; Kakegawa and Yuzaki, 2005). It is not known if this form of pf-PC LTD or the molecules in the downstream pathways are differentially expressed between Purkinje cell subsets.

In contrast, another apparent manifestation of pf-PC LTP has a close relationship to cerebellar stripes. This instance comes from the flavoprotein autofluorescence imaging of cerebellar activity by Ebner and colleagues (e.g., Wang et al., 2009, 2011; Ebner et al., 2012). By stimulating mouse cerebellar cortex by using a paradigm that induces LTP at pf-PC synapses, an array of long-latency patches was revealed that aligns with the zebrin II+ Purkinje cell stripes and shows robust LTP. This form of LTP is mGluR1-dependent and blocked by application of PLCβ and ryanodine receptor inhibitors. This is pertinent because both mGluR1 receptor subtypes (Mateos et al., 2001; *III.ii* above) and PLCβ isoforms (Sarna et al., 2006; *III.vii.* above) are expressed in stripes. How this expression of LTP relates to that described above, is unclear.

## Conclusions

In this review, LTD at the pf-PC synapse has been used as an example of a correlation between the molecular architecture of the cerebellar cortex and the specialization of cerebellar function. To recapitulate, the data show two things: first, LTD is manifested differently in different stripes; and secondly, some of the molecules implicated in the LTD signaling pathways also show expression patterns restricted to stripes, ranging from convincing (e.g., mGluR1b, EAAT4, PLCβ3, PLCβ3/4, GABA_B_R2) to intriguing (e.g., nNOS, CRF), to being of marginal significance at best (e.g., IP_3_R; IGF-1; PKCδ: Table 1). While this review has focused on one aspect of cerebellar function as an exemplar—LTD at the pf-PC synapse—it would be surprising if the molecular architecture were not similarly customized to serve other cerebellar functions. The evidence that LTP at the pf-PC synapse may also vary across stripes is also briefly reviewed. The conclusion is thus that cerebellar function has evolved to accommodate the different requirements of multiple, parallel afferent and efferent pathways, by customizing key molecular constituents. For example, on the afferent side Purkinje cell stripes receive mossy fiber pathway input from multiple sources and with very different firing patterns—have pf-PC synapses specialized to accommodate this? Likewise on the efferent side, do different cortical receiving areas require different LTD kinetics? Another consideration is that perhaps stripes work as zebrin II+/− pairs. One hint that this might be the case comes from the studies of optic flow in the pigeon cerebellum by Graham and Wylie (2012), which show that Purkinje cells in zebrin II+/− stripe pairs all respond best to the same pattern of optic flow. Given that climbing fibers onto zebrin II+ Purkinje cell stripes release more glutamate than those onto zebrin II- stripes (e.g., Paukert et al., 2010) it may be that both slow and fast adapting stripes work in concert as the fundamental functional unit in the cerebellar cortex.

## Conflict of interest statement

The author declares that the research was conducted in the absence of any commercial or financial relationships that could be construed as a potential conflict of interest.
